# Urban Land-Cover Changes in Major Cities in China from 1990 to 2015

**DOI:** 10.3390/ijerph192316079

**Published:** 2022-12-01

**Authors:** Qian Ding, Tao Pan, Tao Lin, Chi Zhang

**Affiliations:** 1 Shandong Provincial Key Laboratory of Water and Soil Conservation and Environmental Protection, College of Resources and Environment, Linyi University, Linyi 270600, China; 2 School of Geography and Tourism, Qufu Normal University, Rizhao 276826, China; 3 Key Laboratory of Urban Environment and Health, Institute of Urban Environment, Chinese Academy of Sciences, Xiamen 361021, China; 4 Research Center for Ecology and Environment of Central Asia, Chinese Academy of Sciences, Urumqi 830011, China; 5 State Key Laboratory of Desert and Oasis Ecology, Xinjiang Institute of Ecology and Geography, Chinese Academy of Sciences, Urumqi 830011, China

**Keywords:** urban expansion, land-cover change, greening, impervious surfaces, China

## Abstract

The accelerated urbanization process in China has led to land-cover changes, triggering a series of environmental issues as one of the major drivers of global change. We studied the land-cover changes in the built-up areas of 50 major cities in China from 1990 to 2015 with Landsat data combined with spectral unmixing methods and decision tree classification. The overall accuracy of urban land-cover type products with 30 m resolution was obtained as 84%, which includes impervious surfaces, bare soil, vegetation, and water bodies. Based on these land-cover type products, the results show that the urbanization of major cities in China manifests itself as a steep expansion of impervious surfaces (+32.91%) and vegetation (+36.93%), while the proportion of bare soil (−68.64%) and water bodies (−1.20%) decreases. The increase in vegetation indicates an increasing emphasis on greening during urbanization, which is especially vital for the sustainability of urban ecosystems. Increasing economic standards and population sizes are significantly correlated with impervious surface expansion and may be the main drivers of urbanization. Nationwide, there is a decreasing trend of shape complexity among different large cities, which indicates that landscape shapes will gradually become regular when cities grow to a certain level. Greenspace areas in the cities increased significantly during 1990–2015 and became more fragmented and tended to disperse across cities. These changes reflect the government’s efforts to enhance urban ecosystem functions to serve the rapidly increasing urban population in China over the past three decades.

## 1. Introduction

A major land-use change in the 21st century is urbanization [1,2]. In the first half of the century, the built-up area is expected to triple [3]. In China, the urbanization rate is twice that of the world, with 67% of the urban space impervious to water, far higher than the average for the world [4,5]. Most previous national-scale studies on urban lands in China focused on the overall urban area [6,7] or a certain urban land-cover type, such as impervious surfaces or greenspaces [8,9], while the detailed land-cover/landscape structures and their evolving patterns across the urbanized areas in China are still unclear.

Recent decades have seen large-scale changes in land-use patterns all over the world, impacting the landscape patterns and functions of metropolitan areas [10,11]. A landscape ecology perspective can be used to measure the evolution of urban systems [12]. The research methods of landscape analysis include landscape indices, statistical analysis of land space, and ecological models of landscapes [13]. On the one hand, urbanization increases the fragmentation and complexity of urban landscapes to some extent in accordance with the aggregation-dispersion hypothesis (urbanization results in landscape patterns with increasing compositional diversity, geospatial complexity and ecological fragmentation), and on the other hand, it has been hypothesized that intensive human management and disturbances could lead to similar landscape patterns and reduced structural complexity of urban ecosystems in cities from regions with very different bioclimatic backgrounds (i.e., the homogenization hypothesis) [14]. It is not clear what kind of landscape patterns are changing in China’s major cities.

The urbanization process is a dynamic change process of human-land relations [15], and urban population density and urban land-use structure cannot be ignored when studying human-land relations in the urbanization [16]. To achieve sustained growth, the Chinese government has proposed supply-side structural reforms [17]. Land is a fundamental resource for participation in socioeconomic activities [15], and structural reform of the supply side of urban land is crucial for optimizing the structure and distribution of elements and attributes of cities. Therefore, it is necessary to study the effects of population density and socioeconomic level on urban land cover dynamics to reveal the response of urban land cover changes to the socioeconomic factors.

These knowledge gaps have severely limited our ability to evaluate the urbanization effects on China’s ecosystem sustainability, considering the high heterogeneity of urban landscapes and the huge differences in ecological structure/functions among different urban land-cover types. Therefore, it is necessary to characterize spatial and temporal changes in urban landscape patterns to elucidate trends in land structure changes in China’s metropolitan areas and to provide insights into the driving forces (e.g., socioeconomic factors) and accompanying ecological impacts that are important for the design of sustainable urban development policies.

**Figure 1:**
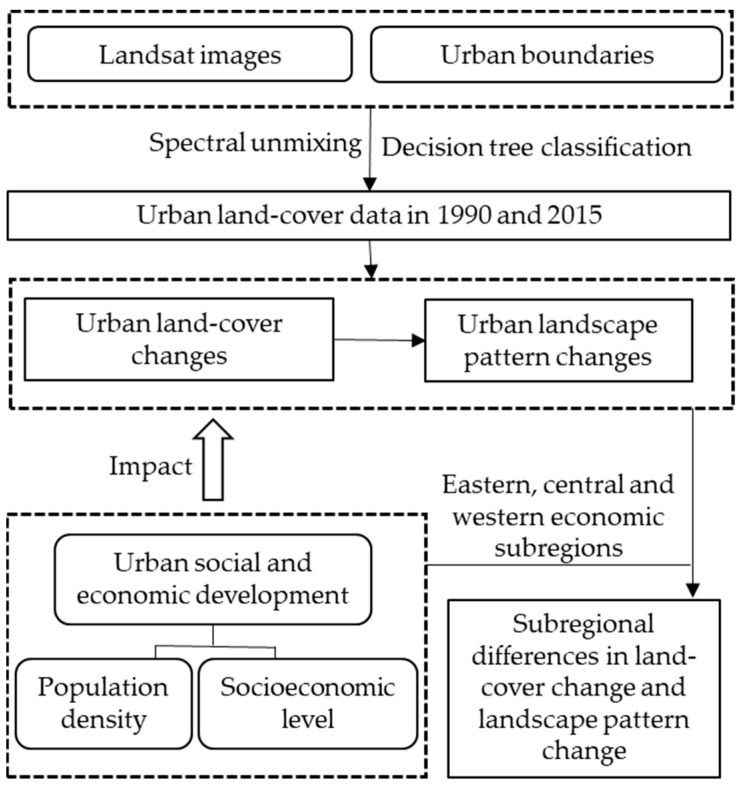
Conceptual framework of the study.

Ensuring the sustainable development goals (SDGs) of China requires detecting the land dynamic patterns in urbanized areas across the nation and answering important questions, including the following: How did the composition and configuration of different land functional types (water, impervious surfaces, greenspaces, and bare soils) evolve over time? How did urbanization respond to the social economy? Therefore, the objectives of this study are (1) to obtain the dynamic characteristics of land cover in large cities in China from 1990 to 2015 using change detection analysis strategy, especially the urban impervious surface, and (2) to explore the response of urban impervious surface change to socioeconomic development (Figure 1).

## 2. Materials and Methods

### 2.1. Study Area

**Figure 2:**
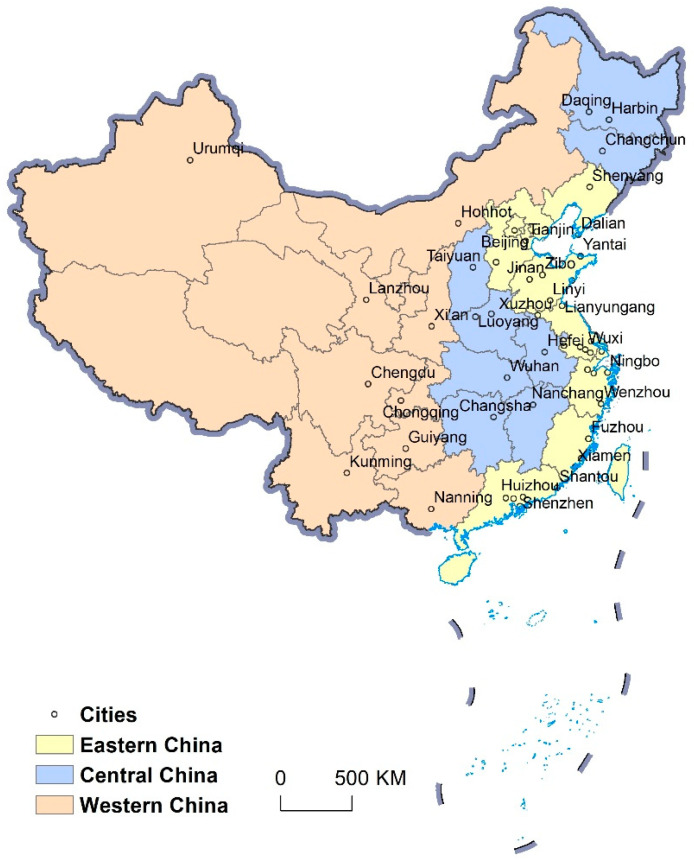
Locations of the 50 major cities in the three subregions of China. (Base on map sources: GS (2019)1651.)

Based on the built-up area of China in 2015 published by the Ministry of Construction of the People’s Republic of China and Urban-Rural Development (https://www.mohurd.gov.cn/ (accessed on 23 April 2021)), the top 50 large cities with built-up areas in 2015 were selected as the research subjects. These cities constitute 43.3% of the overall urban built-up areas in China. Therefore, these 50 cities are representative of urban cover change patterns (Figure 2). The 50 cities are divided into eastern, central and western cities based on their level of economic development.

### 2.2. Land-Cover Mapping

#### 2.2.1. Data Acquisition and Preprocessing

Landsat 5 (TM) and Landsat 8 (OLI) images at 30 m spatial resolution were obtained from the United States Geological Survey (USGS) database for 1990 and 2015, respectively. The Landsat images were geometrically corrected based on topographic maps obtained from the USGS using ENVI 5.3 software. The effect of atmospheric scattering in the images was removed by subtracting pixel values representing the background features in each band of the radiometric correction by dark subtraction correction. Atmospheric correction was carried out to eliminate radiation errors. The areas and proportions of urban land-cover types were counted based on the same projection (Albers) and coordinate system (WGS 1984). Urban built-up area boundaries (1990 and 2015 built-up boundaries, respectively) were determined using the Finer Resolution Observation and monitoring global land cover (http://data.ess.tsinghua.edu.cn/ (accessed on 21 October 2021)).

#### 2.2.2. Urban Land-Cover Classification

Images with medium spatial resolution (30 m) have the advantage of convenience of acquisition and suitable spectral resolution, making them an in-vital and preferable data source for current large-scale land structure studies [18]. Based on their ecological functions, urban land-cover types excluding water bodies can be classifiable into three categories by the vegetation-impervious surface-soil (VIS) model [19,20]. Lu applied the VIS model combined with the linear spectral mixture analysis (LSMA) model for mixing pixel decomposition to extract urban land-cover type information from medium spatial resolution images [21]. Zhang et al. [22] reported a method combining decision tree classification and LSMA to improve the classification accuracy of land-cover types. In this paper, we adopt this method as the basis and combine it with the change detection analysis strategy of Pan et al. [23] to provide accurate and reliable land-cover type information for large cities in China.

The VIS model was used to classify urban land-cover types by combining vegetation, soils, low and high albedo features [23]. Selecting the optimal end member for each component is a critical part of this step in the process. The multispectral bands were converted into three principal components using the least noise fraction conversion method, and then the end elements for the four land-cover types were determined. Landsat images were decomposed into four component results using a fully constrained least squares method [20].

According to the urban VIS model proposed by Ridd [19], the mixed image elements were unmixed by combining linear decomposition. Minimum noise fraction transformation (MNF) was performed to reorganize the information in the initial bands so that the main information was focused in the first three bands [23] and the first three bands were combined two by two to obtain the scatter plot. By this method, only the scatter plot generated from the first three bands is analyzed to obtain the end element, which saves computing time and results in a better scatter plot. Eventually, four end elements were selected: high albedo objects, vegetation, soil, and low albedo objects [24]. The traditional linear decomposition method with an abnormal threshold interval affects the extraction accuracy of the image, but the least squares hybrid decomposition method is used to control the threshold of the extracted components within 0–1, thus improving the extraction accuracy [20], so the original image is decomposed into four-component images using the fully constrained least squares method. Since the image elements are more complex in urban areas, the decision tree approach can better solve the problem that the medium-resolution remote sensing inversion is prone to misclassify urban land classes, so this paper uses the decision tree approach to define the threshold by superimposing the original synthetic image with the help of the normalized difference water index (NDWI), normalized difference vegetation index (NDVI), and image element values of mid-infrared bands to identify urban land-cover types.

#### 2.2.3. Accuracy Evaluation

The accuracy of surface classification was verified using high-resolution Google satellite images (1 m). A total of 100 sample points (50 random points for impervious surfaces, 20 random points for vegetation, 20 random points for bare soil, and 10 random points for water bodies) were randomly selected for each period in of each city to evaluate the classification accuracy.

### 2.3. Detection of Changes

Information on urban land-cover types at different spatial scales can be used to understand land-cover type changes in major cities in China. The landscape pattern index provides a quantitative reflection of the spatial morphological characteristics of urban land-cover types [25]. The detection of changes includes the following aspects. First, information on the land-cover changes of major cities in China was obtained, and the differences between cities in the three sub-regions were analyzed. Second, land-cover changes within the old urban areas were studied. Finally, landscape pattern changes in urban land-cover types were analyzed at national and subregional scales.

### 2.4. Calculation of Landscape Pattern Index

Landscape indices, for which many metrics are available, have been widely used to assess land-cover changes and landscape patterns [26]. The landscape pattern index quantifies the fragmentation of landscape patterns, connectivity, and heterogeneity at the patch, class and landscape levels [27]. Most of the landscape index models were collected in Fragstats 4.2 and are easy to calculate. It is very important to choose the appropriate metrics to analyze the landscape patterns. Patch density (PD) and largest patch index (LPI) metrics were chosen to analyze the landscape components. The PD and LPI metrics quantify characteristics such as area, diversity, and density associated with each land-cover type. Indicators such as aggregation index (AI) and landscape shape index (LSI) are used to explain the configuration of the landscape pattern and landscape characteristics such as shape, spatial distribution, continuity, clustering, and fractality. Overall landscape indices at the land-cover type level and at the national and subregional levels were calculated to comprehensively analyze the landscape pattern of large cities in China.

### 2.5. Effects of Socioeconomic Factors on Urban Land-Cover Changes

Impervious surface area (ISA) is a significant symbol of the transformation from natural to artificial surfaces in the urbanization process, and its transformation changes the proportion of land-cover types in built-up urban areas [28]. We analyzed whether there is a significant correlation between changes in ISA and changes in socioeconomic factors (GDP and population) and tried to develop a corresponding linear model. We explored whether changes in socioeconomic factors have a driving/hindering effect on changes in urban land cover, with a positive correlation representing a positive feedback effect and a negative correlation representing a negative feedback effect. Data for socioeconomic factors (GDP and urban population) were collected from the National Bureau of Statistics of China (http://www.stats.gov.cn/tjsj/tjgb/ndtjgb/ (accessed on 11 May 2022)).

## 3. Results

### 3.1. Urban Land-Cover Type Classification Accuracy

For the 50 major cities in China, the overall classification accuracy of land-cover type classification results in 1990 and 2015 were 83.28 ± 0.04% and 84.28 ± 0.03% (mean ± 1 SD), with kappa coefficients of 0.75 ± 0.05 and 0.78 ± 0.04, respectively (Table 1). At the subregional scale, the overall classification accuracy in 1990 ranged from 82.2 ± 0.02% to 83.75 ± 0.04%, and the kappa coefficient ranged from 0.73 ± 0.02 to 0.75 ± 0.06. At the subregional scale, the overall classification accuracy in 2015 ranged from 82.4 ± 0.03% to 84.68 ± 0.03%, and the range of kappa coefficients was 0.73 ± 0.05 to 0.78 ± 0.04. The overall classification accuracy for all cities was above 80%.

### 3.2. Urban Land-Cover Type Changes

#### 3.2.1. Dynamic Changes in the Characteristics of Urban Land-Cover Types

**Figure 3:**
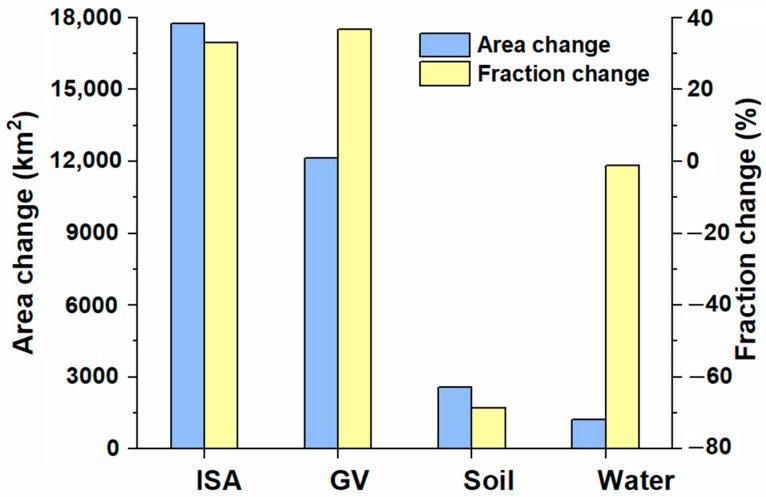
Changes in urban land cover between 1990 and 2015. Abbreviations: ISA: impervious surface area; GV: vegetation.

**Figure 4:**
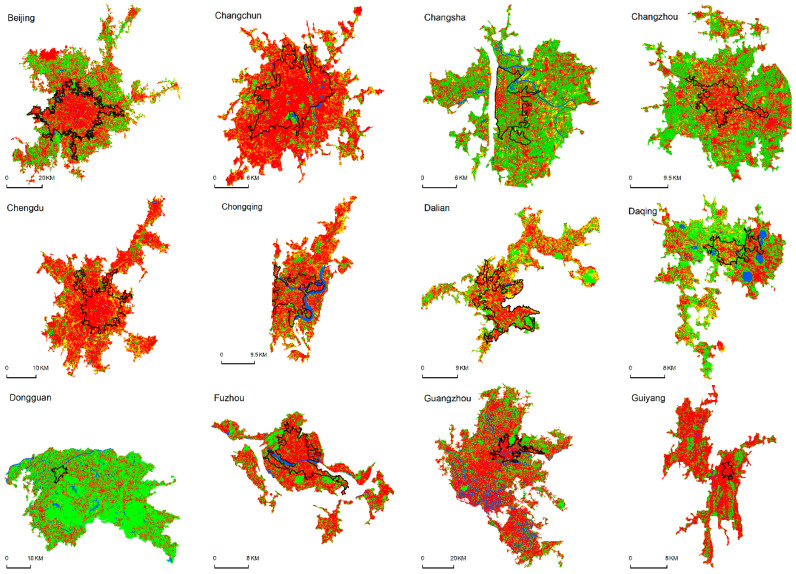

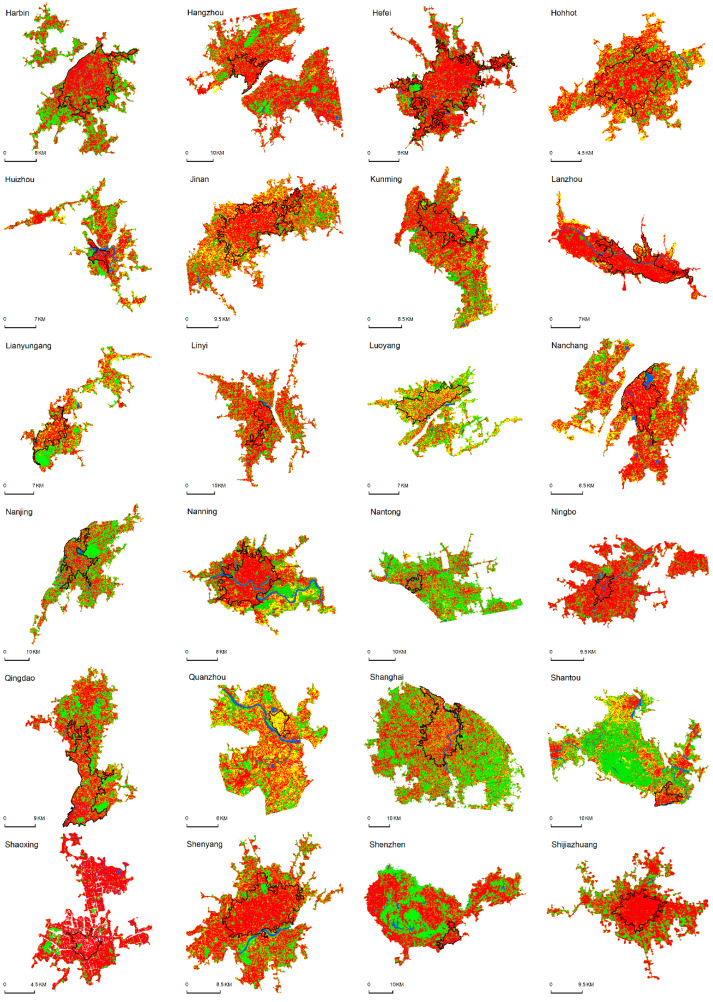

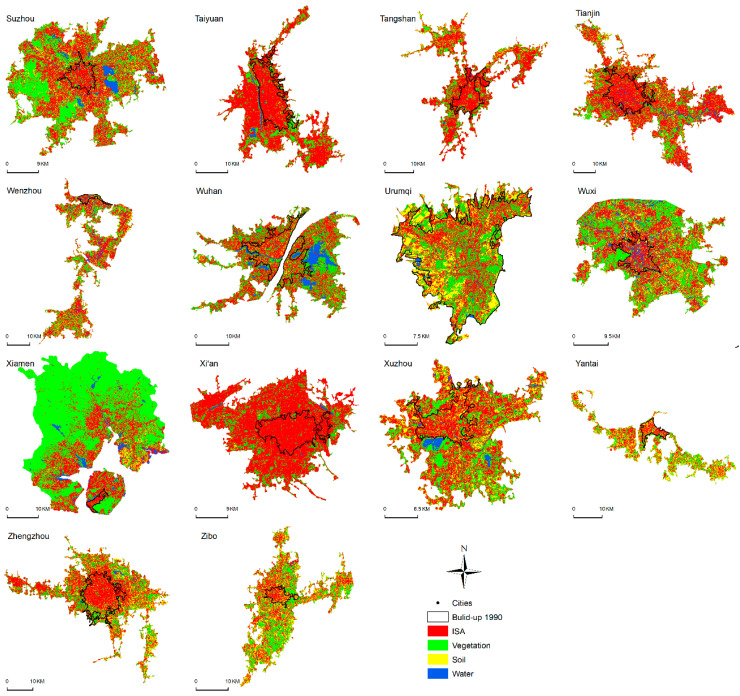
Land cover for the 50 major cities in China in 2015.

The results of the change detection (Figure 3 and Figure 4) show that the urban built-up area increased from 5912 km^2^ to 39,592.86 km^2^ from 1990 to 2015, or an increase of 33,681.09 km^2^. Of this amount, ISA increased by 17,750.31 km^2^ (32.91% in the urban built-up area), which indicates a compact urbanization pattern. The vegetation (GV) increased by 12,145 km^2^ (36.93% in the urban built-up area), which indicates better greening of the urban space. The shrinking of the bare soil (−68.64%) is the most prominent urban land change, while the water bodies remained stable (−1.20%). In addition, urban development was higher in the original urban built-up areas and near the main roads, and many new urban cores were formed along the main roads. (Figure 3).

#### 3.2.2. Changes in Land Cover in Old Urban Areas

Table 2 summarizes the changes in land cover in the old urban areas during the urbanization process. In the old urban area, ISA increased by 1031 km^2^, of which 41% was previously vegetated and 55% was previously bare soil. At the same time, 700 km^2^ of ISA in the old urban area was converted to other types (vegetation, bare soil and water). Moreover, the area converted from bare soil and ISA to vegetation corresponded to 67% of the lost vegetation area; as a result, the proportion of vegetation in the old urban area was reduced by 4.97%. The loss rate of bare soil in the old urban districts is approximately 58.39%, indicating that a large amount of unused land becomes construction land or green space. The changes in water bodies are small.

### 3.3. Spatial and Temporal Changes in Landscape Patterns

**Figure 5:**
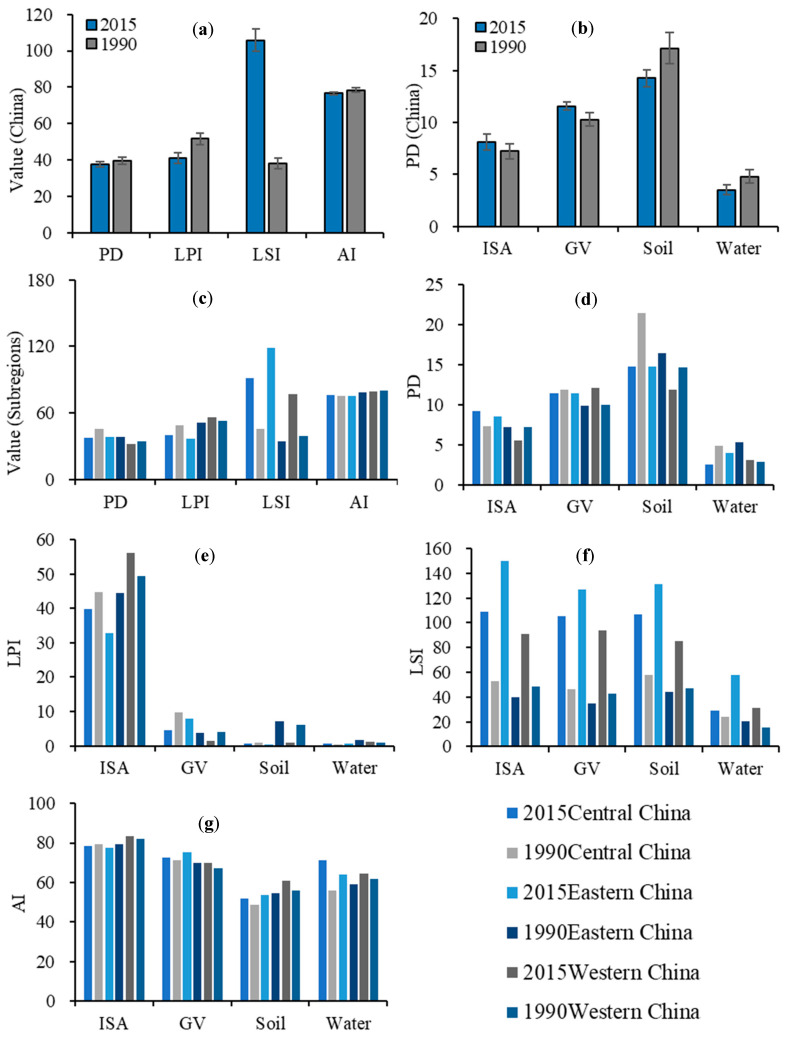
Changes in the landscape indexes from 1990 to 2015. (**a**) Changes in landscape patterns at the national scale. (**b**) Patch density (PD) changes in land-cover types at the national scale. (**c**) Changes in landscape patterns at the subregional scale. (**d**) PD changes in land-cover types at the subregional scale. (**e**) Largest patch index (LPI) changes in land-cover types at the subregional scale. (**f**) Landscape shape index (LSI) changes in land-cover types at the subregional scale. (**g**) Aggregation index (AI) changes in land-cover types at the subregional scale.

Landscape patterns at the national scale, subregional scale and land-cover patch scale were analyzed for 50 large cities between 1990 and 2015 using landscape indices, including PD, LPI, SHDI, AI and LSI. The results show that at the national landscape scale, the degree of spatial heterogeneity of urban landscapes in major cities in China decreased (PD = −2.01), large patches in urban landscapes tended to fragment (LPI = −10.39), and urban landscape patches were more separated (LSI = 67.05) and less aggregated (AI = −1.79) during 1990–2015 (Figure 5a). At the subregional scale, PD decreases in all three subregions, indicating that the urban landscape generally becomes more convergent, with the greatest change in the central subregion (PD = −7.51). Large patches in the landscape tend to fragment in the central (LPI = −8.57) and eastern (LPI = −14.77) subregions, while dominant patches become larger in the western (LPI = 2.65) subregion. The LSI increases in all subregions, indicating a greater separation of urban landscape patches. In addition, the degree of aggregation was lower in the central part (AI = 0.88) and higher in the east (AI = −2.81) and west (AI = −1.26), indicating that the patches of different land-cover types in the eastern and western subregions become more discrete, and the patches of the same land-cover types in the central subregion gather with each other (Figure 5c).

At the land-cover patch scale, the fragmentation of ISA and vegetation decreased, while bare soil and water bodies became becoming more fragmented (Figure 5b). ISA was more fragmented in the central subregion and eastern subregion, but ISA was more integrated in the western subregion. Vegetation was more fragmented, expect in the eastern subregion. The fragmentation degree of bare soil decreased in all subregions (Figure 5d). Large patches of ISA tended to be more fragmented in the central and eastern ISAs, and vice versa in the west. Macro patches of vegetation tended to be fragmented in the central and western parts and vice versa in the east. Large patches of bare soil became larger in all three subregions. Large patches of water bodies became larger in both the central and western subregions, and vice versa in the eastern subregion (Figure 5e). The separation of all land-cover types was greater in all three subregions (Figure 5f). The degree of aggregation of all land-cover types increased in the central and eastern subregions, while it decreased in the western subregion. The degree of aggregation of vegetation was lower in the three subregions. The degree of aggregation of bare soil was lower in the central and western subregions and lower in the eastern subregion. The degree of aggregation of water bodies was lower in all three subregions (Figure 5g).

### 3.4. Impact of Socioeconomic Factors on Changes in ISA

**Figure 6:**
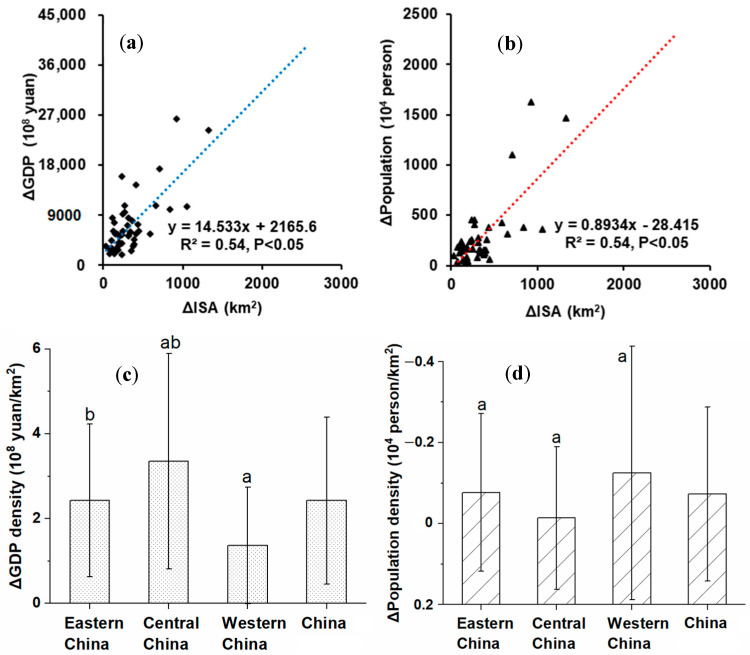
Socioeconomic factors related to ISA changes. (**a**) Correlation between GDP changes and ISA changes. (**b**) Correlation between population changes and ISA changes. (**c**) Changes in national and regional GDP densities. (**d**) Changes in national and regional population densities. The symbol Δ represents a change. The lowercase letters “a” and “b” above the bars in subfigures (**c**,**d**) represent significant differences at 5% level.

The expansion of ISA in China was significantly and positively correlated with the growth of GDP (except for Guangzhou) and the growth population (except for Guangzhou and Chongqing) (Figure 6a,b). Guangzhou experienced rapid ISA expansion (2580 km^2^) from 1990 to 2015. Although the economy (GDP increased by ¥17,839 × 10^8^ Yuan) also developed rapidly, the ratio between its economic development rate and ISA growth rate was relatively low. Moreover, compared with other large cities, Guangzhou’s population growth is relatively small (+497 × 10^4^ person). Chongqing, on the other hand, has a rapidly growing population (1039 × 10^4^ person), far outpacing the construction of its urban infrastructure. The GDP and population densities of major cities increased by 3958 ± 4900% and −1.55 ± 85.43%, respectively, during 1990–2015, which might have driven the rapid urbanization throughout the country (Figure 6c,d).

## 4. Discussion

Reliable information on urban land structure is important for understanding the urbanization characteristics of large cities in China. This study improves the accuracy of land-use classification (>80% for a single city) by linear spectral decomposition and decision tree classification methods, which is higher than the accuracy (75–80%) of other existing large-scale urban datasets [29,30]. This study shows that the rapid expansion of ISA in China was probably driven by the rapid growth of the economy and population in large cities (Figure 4), which was consistent with the research results of Pan et al. (2017), who showed that the ISA expansions in dryland cites of China might be driven by GDP and population [23]. The environmental impacts of urbanization in China over the past 25 years (1990–2015) were closely related to the landscape changes in major cities, where the fractions of impervious surface area and vegetation area increased by 30% to 40%, while the bare soil surface decreased by over 68% and water remained stable (Figure 3). These results differed from the findings obtained by Yan et al. (2015) [20], who found that ISA increased and vegetation decreased in the city of Urumqi. The analysis of land-cover dynamics in Yan et al.’s study was focused on the period from 1990 to 2010 and focused on a single arid city. In a previous study that investigated the dryland cities in China, Pan et al. (2017) found the ISA in urban areas increased by 13.23% and bare soil decreased by 13.41%, while vegetation (+0.27%) and water (−0.10%) remained stable during 2000–2014 [23]. The trend of the changes was similar to our study results, but the rates of the changes was much smaller than ours. Pan et al.’s research focuses on arid areas and most of them are small cities, while our study focused on big cities across China. Therefore, the differences may reflect the influences of city sizes and locations on urbanization pattern. Similarly, we found that the mean fractions of ISA in the major cities of China in 2015 was 59%, which was significantly lower than that (75%) identified in the humid cities of eastern China (including Beijing, Guangzhou, etc.), but closer to the ISA fraction of the arid cities of China (62%) and American cities (56%) (including Chicago, New York, and Los Angeles) [23,31].

Although urbanization may have taken up a large amount of agricultural land and green space outside the city [32], the proportion of green space inside the built-up areas of the city has not decreased sharply. While many previous studies analyzing dynamic urban land-cover characteristics have concluded that urbanization reduces green space based on fixed built-up areas [20,33], the results reported in this paper consider dynamic built-up area changes, analyzed according to the actual built-up area boundaries in each period, and therefore provide more detailed information. The growing green spaces and stable water bodies in the major cities of China show that China has shifted to more sustainable urban development over the past three decades.

At the national scale, we found less landscape heterogeneity among major cities across China, which is in accordance with the results of previous findings [34]. However, at the city level, we found more land fragmentation, esp. for the green spaces. As an approach to improve the human living environment, more green areas are interspersed among impervious surfaces, leading to the tendency of large patches in the urban landscape to be fragmented. The fragmentation of green spaces not only makes them more accessible to the urban population but might also improve the efficiencies of urban ecosystem services [35].

More than half of human population live in urban areas. Policymakers are working hard to improve urban landscape planning and management to enhance urban ecosystem services to city residents while maintaining the city’s socio-economic functions [4]. To reach the sustainable development goals, it is important to understand the evolving pattern of urban landscape structure and evaluate whether the sustainable development policies in the past have changed the pattern of urban landscape development [36]. Such understanding and evaluation rely on large-scale (in both space and time domains) research and inter-city comparisons. Considering the high spatial heterogeneity of the urban landscape, it is important that a study should cover most of the urban regions in the whole study area (for a country or a continent or the whole world). Until recently, most national to global urban landscape studies only focused on describing the overall expansion pattern of urban areas, but having overlooked the evolution of urban landscape structure such as the changes in the composition and configuration of various land-cover types in cities [37]. This gap limits their ability to evaluation the changes in urban land functions and the impacts of sustainable development policies on urban landscape development. Although there were many small-scale studies that have analyzed landscape structure on individual cities, they were unable to provide an overall picture of the urbanization pattern at national to global scale due to the strong the spatial heterogeneity of urban lands [20,23,38]. This study examined the long-term landscape structure changes in the built-up areas of 50 major cities that cover over 43% of the developed areas in China. The urban landscape dynamic patterns revealed by this study are comparable to previous assessments in dryland China and in a few cities in the southern and eastern China, but found more acute land structure changes esp. in the rapid expansion of greenspace (by 36.93%) and fast decrease in the bare soil area (by −68.64%). It reveals a fast and imbalanced landscape changes across the country and among different land-cover types in the developed areas. It also shows that the greenspace areas both in magnitude and in proportion have increased significantly and consistently across the country, thus confirming the effectiveness of the governments sustainable urban development policies.

There are still some limitations and uncertainties in the data and methods of this study. First, there is uncertainty in the detailed characterization of urban land-cover types using medium-resolution data due to the complexity of the urban landscape. The adoption of higher-resolution remote sensing data can improve the classification accuracy and facilitate the extraction of fine urban overlays [39,40,41,42]. In addition, limited by the resolution of Landsat data, the green space types (grassland, shrub, and woodland) were not further subdivided in this study. Finally, it is possible that the built-up area boundaries [43] used in the study may lead to uncertainty in the statistical results within the urban area due to the uncertainty in the data production process as well, despite the manual correction to improve the accuracy before use.

## 5. Conclusions

Landsat data from 1990 and 2015 were used to extract four land-use types of impervious surfaces, bare soil, vegetation, and water bodies for 50 large cities in China using decision tree and spectral decomposition methods. The overall classification accuracy was over 80% for each city. The urbanization of large cities in China was characterized by the growth of impervious surfaces and vegetation, while water bodies stabilized and bare soil decreased. Urban expansion was driven by rapid economic and population growth in major cities, particularly in southern China. Efforts have been taken to make urban development more sustainable, as indicated by the increased urban green spaces with higher accessibility. The rapid increase in urban population density and expansion of ISA require careful urban landscape planning to improve ecological services from limited green space and water bodies.

## Figures and Tables

**Table 1:** Accuracy of land-cover type classification of major cities in China.

Time	Subregions	East	Middle	West	China
1990	OCA *	83.54 ± 0.04%	82.2 ± 0.02%	83.75 ± 0.04%	83.28 ± 0.04%
	Kappa	0.75 ± 0.06	0.73 ± 0.02	0.75 ± 0.05	0.75 ± 0.05
2015	OCA *	84.68 ± 0.03%	82.4 ± 0.03%	84.28 ± 0.03%	84.28 ± 0.03%
	Kappa	0.77 ± 0.04	0.73 ± 0.05	0.77 ± 0.04	0.78 ± 0.04

* OCA: overall classification accuracy.

**Table 2:** Land-cover changes (km^2^) in the old urban areas between 1990 and 2015.

China	ISA	Vegetation	Soil	Water	1990 Total
ISA	2499	409	232	58	3199
Vegetation	833	323	105	15	1276
Soil	794	233	158	21	1205
Water	104	20	7	45	175
2015 total	4230	985	502	139	5855

## Data Availability

Urban built-up area boundaries can be accessed at the Finer Resolution Observation and monitoring global land cover (http://data.ess.tsinghua.edu.cn/ (accessed on 21 October 2021). GDP and urban population) can be accessed at the National Bureau of Statistics of China (http://www.stats.gov.cn/tjsj/tjgb/ndtjgb/ (accessed on 11 May 2022)). All the data presented in this study are available on request from the corresponding author.
